# The Efficacy of Neoantigen-Loaded Dendritic Cell Vaccine Immunotherapy in Non-Metastatic Gastric Cancer

**DOI:** 10.3390/medsci13030090

**Published:** 2025-07-11

**Authors:** Menelaos Papakonstantinou, Paraskevi Chatzikomnitsa, Areti Danai Gkaitatzi, Athanasia Myriskou, Alexandros Giakoustidis, Dimitrios Giakoustidis, Vasileios N. Papadopoulos

**Affiliations:** First University Surgical Department, Papageorgiou General Hospital, School of Medicine, Aristotle University of Thessaloniki, 56429 Thessaloniki, Greece; voula.hatzikomnitsa@gmail.com (P.C.); aretidanaegtz24@gmail.com (A.D.G.); myriskou@gmail.com (A.M.); alexgiakoustidis@gmail.com (A.G.); dgiakoustidis@gmail.com (D.G.); papadvas@auth.gr (V.N.P.)

**Keywords:** gastric cancer, immunotherapy, vaccine, neoantigen-loaded vaccine, dendritic cell vaccine, efficacy

## Abstract

Introduction: Gastric cancer (GC) is the third leading cause of cancer-related deaths worldwide. Even though surgery and chemotherapy are the mainstay of treatment, immunotherapy, and more specifically anti-tumor vaccination, has gained popularity over the past years due to the lower related toxicity and fewer long-term side effects. Dendritic cell (DC) vaccines have been shown to induce tumor specific cytotoxic T-cell (CTL) responses both in vitro and in vivo; however, due to the nature of the disease, resistance to immunotherapy is often developed. Various modifications, such as the implementation of viral vectors, tumor RNA, or even tumor-specific peptides (neoantigens), have been studied as a means to avoid resistance and enhance the effectiveness of the vaccines. In this review, we aim to assess the effects of neoantigen-loaded DC vaccines (naDCVs) on the immune response against gastric cancer cells. Materials and methods: A thorough literature search was conducted on PubMed and clinicaltrials.gov for studies assessing the efficacy of naDCVs against gastric cancer both in vivo and in vitro. The studies were assessed for eligibility by two independent reviewers based on predetermined inclusion and exclusion criteria. The search was completed following the PRISMA guidelines. Results: Eleven studies were included in our systematic review. In five of the studies, the effects of the naDCVs were tested in vitro; in two and in four they were examined both in vitro and in vivo. The in vitro studies showed that the naDCVs resulted in a more robust immune response against the cancer cells in the study groups compared to the control groups. The in vivo studies conducted on mice showed that tumor volume was reduced in the groups treated with the naDCV compared to the untreated groups. What is more, the cytotoxic effect of CTLs against tumor cells was also increased in the vaccine groups. One of the studies was conducted on humans as a phase I study. The results show increased CTL proliferation and cytokine production in the vaccinated group compared to the control, but no difference regarding the tumor size was observed. Conclusions: Neoantigen-loaded DC vaccines can stimulate a strong immune response against specific gastric cancer cell peptides and enhance tumor cell lysis, therefore hindering or even reversing disease progression, offering great potential for the treatment of patients with gastric cancer.

## 1. Introduction

Gastric cancer is the fifth most commonly diagnosed cancer. Its incidence has increased over the last years and today is the third leading cause of cancer-related deaths [1]. The mainstay of gastric cancer treatment is surgical resection with or without adjuvant therapy, including chemotherapy or radiotherapy. Despite radical therapy, the 5-year survival rate of patients with gastric cancer remains low. For this reason, the development of new treatment methods is necessary [2]. The application of immunotherapy has recently shown potential for the treatment of gastric cancer as it can provide many benefits for patient management, such as the absence of treatment resistance, better survival, and lower toxicity compared to traditional chemotherapy [3].

The Cancer Genome Atlas (TCGA) Research Network has described four groups of gastric cancer based upon molecular classifications, including EBV (Epstein–Barr virus), MSI (microsatellite instability), GS (genomically stable), and CIN (chromosomal instability) [4]. The immune checkpoints of the EBV and the MSI subgroups in particular can serve as potential targets of immunotherapy drugs [5]. The immune system plays an important role in recognizing and eliminating cancer cells, and therapeutic interventions using the immune system in cancer treatment have made significant progress in recent years [6]. To date, these strategies include immune checkpoint blockade (CPB), adoptive cell transfer (ACT), and tumor vaccines [6,7,8,9].

In 2010, the first autologous cancer vaccine based on DC, sipuleucel-T (Provenge), was approved by the U.S. Food and Drug Administration (FDA) for the treatment of prostate cancer [10,11]. To date, this is the only DC vaccine approved by the FDA for the treatment of cancer. There are several studies on the use of DC vaccines for the treatment of other malignancies, such as melanoma, glioma, colorectal, and ovarian cancer [12,13,14]. However, the research on the role of DC vaccines in the management of gastric cancer is still in its early stages, but can potentially offer significant advantages for patients.

DC vaccines are based on the mechanism of dendritic cells, which are the main antigen-presenting cells (APCs), specialized for initiating and regulating immune response [15]. Dendritic cells can recognize antigens produced by cancer cells (neoantigens) and subsequently present them to T lymphocytes located in the lymph nodes that drain the tumor. This process generates cytotoxic T lymphocytes (CTLs) that specifically target the tumor [16]. Additionally, dendritic cells can activate NK cells as well as B lymphocytes, thereby enhancing humoral immunity [3]. Numerous studies on mice have shown that dendritic cells loaded with tumor antigens can induce significant anti-neoplasmatic responses and generate therapeutic immunity against existing tumors [17,18,19,20].

The aim of the present study is to present the current literature regarding the development and effectiveness of neoantigen-loaded DC vaccines (naDCVs) against gastric cancer cells and evaluate their potential role as an adjuvant treatment for patients with gastric cancer.

## 2. Materials and Methods

### 2.1. Search Protocol

A thorough literature review was conducted on PubMed and clinicaltrials.gov for studies regarding the use of neoantigen-loaded DC vaccines against gastric cancer. The terms “gastric cancer”, “advanced gastric cancer”, “vaccine”, “vaccination”, “dendritic cell vaccine”, and “dendritic” were used interchangeably in PubMed and the search yielded 74 results. After excluding 31 duplicates and 15 irrelevant records, 28 underwent further assessment. Thirteen records were excluded after title and abstract screening and fifteen were eligible for full-text screening. Four were excluded as the investigated vaccinations did not meet our inclusion criteria. Finally, 11 records were included in our review.

The clinicaltrials.gov search yielded 7 results after using “gastric cancer” as the condition and “dendritic cell”, “dendritic cell vaccine”, “vaccine”, and “vaccination” as other terms in various combinations. None of the 7 records had any results published, therefore they were excluded from our study.

**Figure 1:**
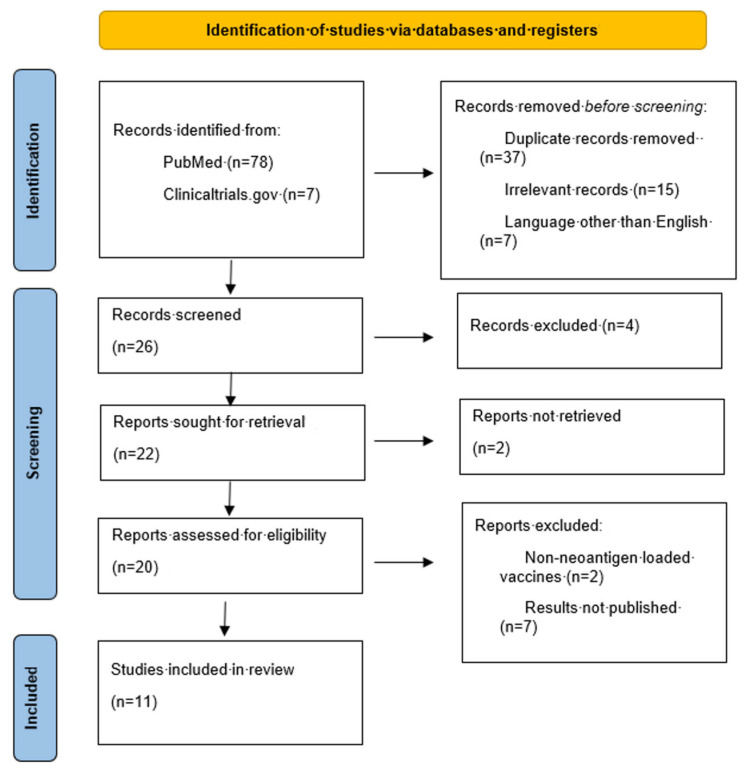
PRISMA flowchart.

The initial search was conducted by two reviewers independently and the databases were last accessed on 14 February 2025. All data were extracted to a predetermined electronic datasheet and were validated by a third reviewer. The following data were extracted: publication year, country, type of study, population, sex, age, cancer cell line, DC collection, neoantigen type, neoantigen–DC fusion process, CTL clone recognition process, vaccine target, dosage, timing, frequency and route of administration, study groups, and results regarding the effectiveness of the vaccines against gastric cancer (such as tumor killing rate, production of tumor-specific CTL clones, cytokine levels, tumor growth and survival). Any conflict during the study selection process was resolved by consulting a senior reviewer. The search was completed according to the Preferred Reporting Items for Systematic Reviews and Meta-Analysis (PRISMA) checklist. The PRISMA flowchart is shown in Figure 1 [21]. The research protocol was registered at the International Prospective Register of Systematic Reviews (PROSPERO, ID CRD420251056722).

### 2.2. Inclusion and Exclusion Criteria

In the present review, we included original, experimental, preclinical, or clinical studies regarding vaccination against non-metastatic gastric cancer written in the English language. The vaccines should be developed after the fusion of DCs with tumor neoantigens and tested either in vivo or in vitro.

The following exclusion criteria were applied: case reports, metastatic gastric cancer, neoantigens derived from cell lines other than gastric cancer, non-neoantigen-loaded DC vaccines, and studies in a language other than English.

### 2.3. Risk of Bias and Quality Assessment

We used the SYRCLES risk of bias assessment tool and the CAMARADES quality assessment checklist to evaluate the included studies [22,23]. The data presented in each study were for intermediate risk of bias and of intermediate to good quality. The relevant tables are available as online Supplementary Materials.

## 3. Results

Eleven reports assessing the effectiveness of neoantigen-loaded DC vaccines (naDCVs) against gastric cancer both in vitro and in vivo were included in this review [16,24,25,26,27,28,29,30,31,32,33]. The majority (n = 7) of them were conducted in China, while three were conducted in Japan and one in Iran. In five of the studies, the experiments were completed entirely in vitro, in two they completed in vivo, and in four studies the efficacy of the naDCV was tested both in vitro and in vivo. One of the in vivo studies was a phase I clinical study conducted on humans, while five were conducted on mice (Table 1). A variety of tumor neoantigens derived from either human or murine gastric cancer cells were utilized for the development of the DC vaccines and are presented in Table 2.

The DCs were collected from either the peripheral blood, bone marrow, or spleen of humans or mice. Peripheral blood mononuclear cells (PBMCs), bone marrow dendritic cells, or spleen cells were cultured with granulocyte–macrophage colony stimulating factor (GM-CSF) and cytokines (e.g., interleukin 4 or TNFa) for 4 to 8 days and the DCs derived were incubated with the tumor-specific neoantigens that are mentioned in Table 2. A variety of incubation methods were reported across the included studies that all led to the fusion of DCs with the tumor neoantigens. Dendritic cells were exposed to neoantigens for several hours up to one week, and the fused DCs were then used for vaccination. Details on the DC collection method and the duration of incubation are shown in Table 3. Additionally, cytotoxic T lymphocytes (CTLs) were collected from peripheral blood from murine or healthy human donors and incubated with peptide-pulsed DCs from 2 h up to 14 days in cultures with GM-CSF and cytokines. Their activity against tumor-specific antigens was then determined with tetrazolium (MTT), chromium-51 (51Cr) release, or cell counting kit 8 (CCK8) assays, or by measuring cytokine levels (IFN-γ, TNFα, IL-2) with ELISA (Table 4). Finally, the characteristics of the DC vaccines used in vitro and vivo are shown in Table 5 and Table 6, respectively.

In nine studies, in vitro vaccines were developed and targeted against gastric cancer cells (Table 5) [16,24,25,26,27,29,30,32,33]. The DCs were pulsed with tumor-specific neoantigens as described previously, and in four of the studies, a vaccine vector was also utilized. Viral vectors (adenovirus or lentivirus) were used in three studies while a synthetic vector (polylactic co-glycolic acid nanoparticle, PLGA) was used in one. In six studies, naDCVs were tested in vivo (mice or humans) [16,28,29,30,31,33]. Viral (lentivirus, or adenovirus) or synthetic (DOTA-P) vectors were used in three studies. All vaccines consisted of pulsed DCs targeting murine or human gastric cancer cells. The dosage was uniform in four studies (10^6^ DCs), while in two studies, the vaccination dosage was 10^7^ DCs and 25 μg of DCs, respectively. The vaccination scheme, however, varied among the studies and ranged from 1 to 6 doses repeated in variable intervals (Table 6). The vaccines were administered either intravenously, subcutaneously, intraperitoneally, or intradermally.

In 9 out of the 11 included studies, the effect of the naDCV was tested in vitro [16,24,25,26,27,29,30,32,33]. The neoantigen-loaded DC vaccinations resulted in a more robust immune response against the cancer cells in comparison to the control groups in all of the included studies, and in five of them, the difference was statistically significant. The magnitude of the immune response was determined via the CTL’s killing rate and the levels of IFN-γ produced. Again, in five of the studies, the IFN-γ levels produced after the neoantigen-loaded vaccinations were statistically significantly higher compared to the control groups (Table 7).

In 6 out of the 11 included studies, the effect of the naDCV was tested in vivo [16,28,29,30,31,33]. In five of the studies, the vaccines were administered to mice inoculated with gastric cancer cells. The tumor volume was reduced, or its growth rate was significantly reduced in the groups treated with the naDCV compared to the untreated groups. The cytotoxic CTL effect against tumor cells was also increased in comparison to the control groups. Of note, in the study of Lu et al., the naDCVs were administered to human patients with advanced-stage gastric cancer, and the vaccine group demonstrated increased CTL proliferation and cytokine production; however, no effect on the size of the tumor was observed. They reported that patients with stable disease had significantly longer survival than those with progressive disease (*p* < 0.05). The results of each individual study are shown in Table 8.

## 4. Discussion

In this systematic review, we evaluated the effectiveness of naDCVs against gastric cancer based on the existing literature. The majority of the published studies tested the in vitro cytotoxic effects of the CTLs activated by the naDCV. The results are uniform among the various studies; the CTLs that were co-cultured with neoantigen-loaded DCs result in greater cytotoxic effects compared to non-specific CTLs. Obviously, the exposure of innate immunity cells to neoantigen-presenting DCs can lead to a significant immune response against gastric cancer cells. This is prevalent by the significantly higher IFN-γ and IL-12 levels detected in the study groups compared to the controls. Of note, as shown in the study of Iwauchi et al., exposure to higher peptide concentrations led to more statistically significant results [26]. This may imply that the magnitude of the effect of the immune response could be proportional to the presented peptide concentration. Similarly, Song et al. concluded that a higher target cell-to-effector cell ratio results in a stronger tumor killing rate [25]. Kohnepushi et al. used PLGA nanoparticles to encapsulate the tumor neoantigen (lysate) and created three groups. One group was exposed to DCs pulsed with lysate encapsulated in nanoparticles, another group was exposed to DCs pulsed with soluble lysate, and a control group was exposed to DCs pulsed with blank nanoparticles. They showed that the tumor specific immune response in the control group was significantly lower than both the other two exposed groups, which further validates the role of tumor-specific CTLs [27].

Six of the studies assessed the effectiveness of naDCVs on tumor growth and survival in vivo. Five of them involved mice (with induced gastric cancer) and one was performed on humans with only minor adverse events. The results on the survival of mice were not conclusive, however a survival benefit could be seen when a neoantigen-loaded vaccine was administered. In the human study, patients with stable disease had a significantly longer survival than those with progressive disease, regardless of the vaccination. The lowest survival rate was observed in two patients with no immunological response. Unfortunately, the human vaccine failed to demonstrate any effect on tumor size or tumor growth, even though tumor-specific CTL proliferation, cytotoxicity, and cytokine production were increased in the study group compared to the control [33]. This may be due to the small number of participants (n = 10) or the vaccination schedule (1 or 2 doses in total per patient) in this phase I study. On the contrary, in all mice studies, the tumor size was significantly reduced in the groups treated with naDCVs. To our knowledge, this is the first systematic review to summarize the current data on neoantigen-loaded DC vaccines against gastric cancer. Their efficacy has been well-demonstrated against other types of cancer, such as prostate, lung, and breast cancers [34,35,36]. However, due to the unique molecular characteristics of gastric cancer, meticulous research on neoantigen pulsed DC vaccination has not yet been conducted [37].

Dendritic cell vaccines have advantages compared to other immunotherapy vaccines, as they demonstrate good tolerance and high safety for the patient [38]. However, due to the action of dendritic cells in immune response, the recognition and presentation of the antigen to dendritic cells play crucial roles, which hinders the development of a dendritic cell-based vaccine [39]. The antigens used to activate dendritic cells are DNA, synthetic peptides, whole tumor RNA, as well as products from the lysis of tumor cells. Occasionally, entire cells derived from the tumor can also be used [40]. By utilizing tumor-specific antigens, which are not present in normal cells, and therefore not present in the thymus, the neoantigen-loaded DC vaccination overcomes the immune tolerance barrier that many other immunotherapy regimens confront [41]. Due to the difficulty of isolating specific antigens capable of triggering a strong immune response, the creation of DC vaccines becomes challenging and their costs increase. Additionally, these vaccines should contain the smallest possible dose of targeted dendritic cells that will elicit a strong immune response against cancer [42].

Gastric cancer is characterized by great heterogeneity, and the effectiveness of immunotherapy varies among patients. Despite these challenges, DC vaccines present considerable hope for improving treatment if the obstacles related to their creation and safety can be addressed. The safety of DC vaccines has been examined in numerous studies, with side effects primarily including fever, fatigue, and lymphadenopathy. A comprehensive meta-analysis of DC vaccination for prostate cancer and kidney cancer indicated that these vaccines induced tumor-specific immune responses in 77% and 61% of vaccinated patients, respectively [43]. However, DC vaccines are not equally effective in all cancer types. Cancer cells develop unique immune escape mechanisms that may interfere with both innate as well as adaptive immunity [44]. The immunosuppressive tumor microenvironment is a core reason for the limited effectiveness of DC vaccination, since it has the ability to inhibit the function of DCs [41]. Nevertheless, our continuously growing understanding of tumor biology and microenvironment has shown evidence that vaccine effectiveness appears to improve when co-administered with other forms of immunotherapy [45].

Due to the heterogeneity of gastric cancer biology and the variable response to immunotherapy, many predictive biomarkers have been proposed to identify the group of patients that would benefit the most from each treatment modality. Neoplastic angiogenesis is a well-described process in cancer progression, and micro-RNAs (miRNAs) are crucial components of neoangiogenesis gene expression. Giuppi et al. attempted to identify the various miRNAs involved in gastric cancer progression molecular pathways and highlighted their potential role as prognostic markers of survival and clinical response to therapy [46]. Many other potential biomarkers that interfere with tumor immunity by regulating the immunosuppressive microenvironment have been studied, such as inflammation-related genes, ferroptosis-related genes, and lactate metabolism-related genes [47,48,49]. These genes may serve as useful predictors of survival and of responses to immunotherapy for patients with gastric cancer; therefore, future research should focus on identifying key genes that can be effective immunotherapy targets. Differentially expressed genes of T cells and glycosyltransferase have also been proposed as markers for targeted therapy [50,51]. However, the results are not yet conclusive, since most researchers have developed risk assessment models, which are not widely validated in clinical practice. Advances in molecular biology and advanced sequencing techniques, such as Next Generation Sequencing (NGS), will hopefully help identify optimal immune response and prognostic biomarkers that could potentially lead to personalized treatment strategies for patients with gastric cancer.

Our review has limitations that need to be acknowledged. Firstly, each study investigated a DC vaccine pulsed with different neoantigens. Even though all can be effective, it is not possible to choose the naDCVs with the best performance. In vivo vaccinations differed in terms of pulsed DCs dosage, timing, frequency, and even route of administration. Enhancers and vaccine vectors were also used in some studies. Regarding the development of the pulsed DCs and peptide-specific CTLs, even though the basis of inoculation and culture was performed with similar stimulating factors, multiple media were used and the timing of co-culturing differed among all studies. It is obvious that the research is still in the early stages and various research teams develop their own protocols. Only one phase I clinical trial was available with published results, which has shown potential, however phase II and III trials are needed to evaluate the clinical effectiveness of a naDCV on humans.

Future research should focus on standardizing pulsed DC vaccine development and determining if it is possible for gastric cancer neoantigen to induce the best tumor killing effect. After validating the safety with multiple phase I studies, the clinical effects should then be evaluated with phase II and large multicentered phase III trials. Finally, vaccination alone may not be enough since tumors have the potential to evade immunity. Multi-adjuvant approaches that combine both vaccination, immune system stimulation, and immune inhibition prevention should be explored. Looking ahead, the goal for treating gastric cancer is to identify the optimal immunological pathways, where the combined use of immunotherapy and DC vaccines can yield the optimal outcomes for patients.

## 5. Conclusions

In the present systematic review, we assessed the role of naDCVs on immune response and tumor growth against gastric cancer cells both in vitro and in vivo. Vaccination with neoantigen-loaded DCs results in a peptide-specific CTL response, which then leads to increased cytotoxicity against tumor cells and enhanced cytokine production. What is more, a significant reduction in tumor size was observed in all animal studies. In the phase I human study, the naDCVs were safe with minor toxicity and enhanced the peptide-specific immune response, but did not affect the tumor size. The results are promising, however the research on humans is still in its early stages. In the future, more clinical trials, especially phase II and large multicentered phase III trials, are needed in order to thoroughly evaluate the clinical efficacy of this promising adjuvant gastric cancer treatment.

## Figures and Tables

**Table 1:** Study characteristics.

Study	Year	Country	Study Type	Population
Cai [24]	2007	China	in vitro	NA
Song [25]	2012	China	in vitro	NA
Iwauchi [26]	2010	Japan	in vitro	NA
Kohnepoushi [27]	2019	Iran	in vitro	NA
Liu [28]	2004	China	in vivo	Mice
Wang [29]	2015	China	in vitro and in vivo	Mice
He [30]	2010	China	in vitro and in vivo	Mice
Nagaoka [31]	2021	Japan	in vivo	Mice
Zhu [16]	2018	China	in vitro and in vivo	Mice
Nabeta [32]	2000	Japan	in vitro	NA
Lu [33]	2018	China	in vitro and in vivo	Humans

NA, not applicable.

**Table 2:** Neoantigens used for the development of DC vaccines.

Study	Neoantigen	Derived From
Cai [24]	Heparanase	Plasmid pcDNA3-Hpa
Song [25]	total RNA from MFC/4-1BBL cells	MFC
Iwauchi [26]	ERas peptide (ERas-A24-120)	Human scirrhous gastric cancer cells (OCUM-8, OCUM-2MD3, OCUM-2M)
Kohnepoushi [27]	Tumor lysate	Human gastric cancer cells
Liu [28]	Tumor total RNA	MFC
Wang [29]	MG7-Ag	Human gastric cancer cells
He [30]	MAGE-1	Human gastric cancer cells
Nagaoka [31]	mCdt1, mScarb2, mZfp106	YTN2 and YTN16 murine gastric cancer cells
Zhu [16]	MG7-Ag	Human gastric cancer cells
Nabeta [32]	F4.2 peptide	Signet ring gastric cancer cells
Lu [33]	WT-1 peptide	Human gastric cancer cells

pcDNA3-Hpa, plasmid containing the full-length DNA of heparanase; MFC/4-1BBL cells, MFC cell line transfected with the plasmid pMKITneo/4-1BBL; MFC, murine forestomach carcinoma; MG7-Ag, monoclonal gastric cancer 7 antigen; MAGE-1, melanoma antigen gene 1; WT-1, Wilms tumor protein-1.

**Table 3:** The development of pulsed DCs.

Study	Collection of the DCs		Fusion of the DCs	
	Culture with GM-CSF	Duration	Neoantigen	Duration
Cai [24]	PBMCs	5 days	rAd-Hpa	3 days
Song [25]	Murine BMDCs	5 days	MFC/4-1BBL RNA	7 days
Iwauchi [26]	PBMCs	5 days	ERas HLA-A*2402	5 days
Kohnepoushi [27]	PBMCs	4 days	Tumor lysate	4 days
Liu [28]	Spleen cells	24 h	DOTAP and total RNA	2–4 h
Wang [29]	BMDCs	6 days	T7-MG1 or T7-MG3	24 h
He [30]	Murine PBMCs	8–9 days	rAd-MAGE-1	2 h
Nagaoka [31]	Murine BMDCs	8 days	mCdt1, mScarb2, mZfp106	2 h
Zhu [16]	Human PBMCs	5 days	MG-7	7 days
Nabeta [32]	Human PBMCs	4–7 days	F4.2	2 days
Lu [33]	Human PBMCs	5 days	WT1	30 min

DCs, dendritic cells; PBMCs, peripheral blood mononuclear cells; GM-CSF, granulocyte–macrophage colony stimulating factor; rAd-Hpa, recombinant adenovirus encoding heparanase; BMDCs, bone marrow dendritic cells; MFC/4-1BBL, MFC cell line transfected with the plasmid pMKITneo/4-1BBL; DOTAP, cationic lipid 1,2-dioleoyl-3-trimethylammonium-propane; T7, toll-like receptor-7 agonist; MG1: monoclonal gastric cancer 7 (MG-7) antigen mono-epitope (peptide sequence is KPHVHTK); MG3: monoclonal gastric cancer 7 (MG-7) antigen tri-epitope (peptide sequence is KPHVHTKPHVHTKPHVHTK); rAd-MAGE-1, recombinant adenovirus encoding melanoma antigen gene 1.

**Table 4:** The development of neoantigen-specific CTLs.

Study	Collection of CTLs	CTL Culture with GM-CSF	Duration (Days)	Identification of CTLs Activity	Identification of IFNγ Levels
Cai [24]	Human peripheral blood lymphocytes	rAd-Hpa pulsed DCs	21 days	51Cr	ELISA
Song [25]	Murine spleen	MFC/4-1BBL/DCs	5 days	MTT	ELISA
Iwauchi [26]	PBMCs	ERas HLA-A*2402 DCs	NS	51Cr	NS
Kohnepoushi [27]	PMBCs	Tumor lysate pulsed DCs	NS	MTT	NS
Liu [28]	Spleen	DOTAP and RNA pulsed DCs	5 days	51Cr	NS
Wang [29]	Spleen	EAC tumor cells	4 h	LDH method	ELISA
He [30]	Spleen	DC-Ad-MAGE-1	14 days	MTT	NS
Nagaoka [31]	Spleen	Irradiated tumor cells	NS	NS	ELISA
Zhu [16]	Spleen	MG-7 pulsed DCs	48 h	CCK-8	NS
Nabeta [32]	Human PBMCs	F4.2 pulsed DCs	2 h	51Cr	NS
Lu [33]	PBMCs	WT1 pulsed DCs	NS	Flow cytometry	ELISA

CTLs, cytotoxic T lymphocytes; GM-CSF, granulocyte–macrophage colony stimulating factor; rAd-Hpa, recombinant adenovirus encoding heparanase; DCs, dendritic cells; 51Cr, chromium-51 release assay; ELISA, enzyme-linked immunosorbent assay; MFC/4-1BBL/DCs, DCs pulsed with MFC cell line transfected with the plasmid pMKITneo/4-1BBL; MTT, dimethylthiazol diphenyl tetrazolium bromide (yellow tetrazolium dye) assay; NS, not specified; PBMCs, peripheral blood mononuclear cells; DOTAP, cationic lipid 1,2-dioleoyl-3-trimethylammonium-propane; EAC, Ehrlich ascites carcinoma; LDH, lactate dehydrogenase; DC-Ad-MAGE-1, DC pulsed with recombinant adenovirus encoding melanoma antigen gene 1; CCK-8, cell counting kit 8 assay.

**Table 5:** The characteristics of in vitro vaccines.

Study	Vaccine Type	Vector	Neoantigen	Target
Cai [24]	DC vaccine	rAd-Hpa	Heparanase	Gastric cancer KATO-III cell line
Song [25]	DC vaccine	pMKITneo/4-1BBL plasmid	Total RNA from MFC/4-1BBL cells	MFC cells
Iwauchi [26]	DC vaccine	None	ERas-A24-120 peptide	Scirrhous gastric cancer cells
Kohnepoushi [27]	DC vaccine	PLGA nanoparticles	Tumor lysate	Gastric cancer cells
Wang [29]	DC vaccine	None	MG7-Ag	EAC cells
He [30]	DC vaccine	Ad-MAGE-1	MAGE-1	Gastric cancer cells
Zhu [16]	DC vaccine	Lentiviral vector encoding MG-7Ag	MG7-Ag	Gastric cancer cell lines KATO-III and MKN45
Nabeta [32]	DC vaccine	None	F4.2 peptide	Gastric cancer cells
Lu [33]	DC vaccine	None	WT-1 peptide	Gastric cancer cells

DC, dendritic cell; rAd-Hpa, recombinant adenovirus encoding heparanase; MFC/4-1BBL, cell line transfected with the plasmid pMKITneo/4-1BBL; MFC, murine forestomach carcinoma; PLGA, polylactic co-glycolic acid; MG7-Ag, monoclonal gastric cancer 7 antigen; EAC, Ehrlich ascites carcinoma; Ad-MAGE-1, recombinant adenovirus encoding MAGE-1; MAGE-1, melanoma antigen gene 1; WT-1, Wilms tumor protein-1.

**Table 6:** The characteristics of in vivo vaccines.

Study	Vaccine Type	Vector	Neoantigen	Target	Enhancer	Dosage (No. of DCs)	Timing	Route of Administration
Liu [28]	DC vaccine	DOTAP-mediated RNA	Tumor total RNA	Gastric cancer cells	NS	1 × 10^6^	Day 0 and day 7	SC
Wang [29]	DC vaccine	None	MG7-Ag	EAC cells	TLR7 agonist	25 μg	Week 2, 4, 6, 9	IP
He [30]	DC vaccine	Ad-MAGE-1	MAGE-1	Gastric cancer cells	CCL3 and CCL20	1 × 10^6^	Day 5 and day 12	SC
Nagaoka [31]	DC vaccine	None	mCdt1, mScarb2, mZfp106	Gastric cancer cell lines YTN2 and YTN16	LPS	1 × 10^6^	Day 5	SC
Zhu [16]	DC vaccine	Lentivirus encoding MG-7Ag	MG-7Ag	Gastric cancer cell lines KATO-3 and MKN45	Polybrene	1 × 10^6^	Once daily for three consecutive days	IV
Lu [33]	DC vaccine	None	WT-1 peptide	Gastric cancer cells	OK-432	1 × 10^7^	Every 2 weeks for at least 6 sessions	ID

DC, dendritic cell; SC, subcutaneously; MG7-Ag, monoclonal gastric cancer 7 antigen; MG3, monoclonal gastric cancer 7 antigen tri-epitope; TLR7, toll-like receptor-7; IP, intraperitoneally; Ad-MAGE-1, recombinant adenovirus encoding MAGE-1; MAGE-1, melanoma antigen gene 1; LPS, lipopolysaccharide; MG7-Ag, MG7 antigen; IV, intravenously; WT-1, Wilms tumor peptide-1; ID, intradermally.

**Table 7:** Results of in vitro vaccinations.

Study	Groups	Vaccination Cytotoxic Effect	IFNγ Levels
Cai [24]	Group A: DC/rAd-Hpa Group B: DC/rAd-LacZ (control) Group C: LC/IL-2 (control)	Group A vs. Group B vs. Group C: 60% vs. 30% vs. 10%	Significantly increased in Group A (*p* < 0.05)
Song [25]	Group A: MFC/4-1BBL/DC Group B: Control group	Group A vs. Group B target to effector ratio killing rate: 1:20 → 53.3% vs. 34.2% 1:10 → 32.1% vs. 26.7% 1:5 → 21.8% vs. 14.7% (*p* < 0.05)	Group A: 9.45 pg/mL; Group B: 5.97 pg/mL (*p* < 0.05)
Iwauchi [26]	Group A: CTLs induced by ERas-pulsed DCs Group B: CTLs induced by non-pulsed DCs	Significantly stronger in Group A (*p* < 0.05)	Significantly higher in Group A (*p* < 0.05)
Kohnepoushi [27]	NP + Ag Group: DCs pulsed with tumor lysate encapsulated in PLGA nanoparticles Ag Group: DCs pulsed with soluble tumor lysate Control: DCs pulsed with blank PLGA nanoparticles	Stronger in the NP + Ag Group (*p* < 0.05)	Significantly higher in the NP + Ag Group (*p* < 0.05)
Wang [29]	Group A: T7-MG1 Group B: T7-MG3 Group C: PBS control	Group A vs. Group C: 40.92% vs. 16.29 (*p* < 0.01)	Higher in Group A vs. Group B (*p* > 0.05)
He [30]	Study Group: DC-Ad-MAGE-1 Adenoviral control Group: DC-Ad-LacZ Tumor lysate Group: DC-MFC Ag Control Group: Untreated DCs	Higher in the Study Group compared to Control Groups	Significantly higher in Study Group (*p* < 0.05)
Zhu [16]	Study Group: DC-MG-7Ag Negative control group: lentiviral vector without MG-7Ag Control group: untreated DCs	Increased in Study Group compared to Control Groups (*p* > 0.05)	NS
Nabeta [32]	Group A: F4.2-pulsed DCs Group B: Unpulsed DCs	10 times greater in Group A vs. Group B	NS
Lu [33]	Group A: WT1-pulsed DCs Group B: no DC vaccine	Greater cytotoxicity in Group A (*p* > 0.05)	Enhanced in Group A (*p* > 0.05)

rAd-Hpa, recombinant adenovirus encoding heparanase; rAd-LacZ, recombinant adenovirus encoding the LacZ protein; DC, dendritic cell; MFC/4-1BBL/DCs, DCs pulsed with MFC cell line transfected with the plasmid pMKITneo/4-1BBL; MFC, murine forestomach carcinoma; CTLs, cytotoxic T lymphocytes; NP, nanoparticle; Ag, antigen; PLGA, poly lactic-co-glycolic acid; MG1: monoclonal gastric cancer 7 antigen mono-epitope (peptide sequence is KPHVHTK); MG3: monoclonal gastric cancer 7 antigen tri-epitope (peptide sequence is KPHVHTKPHVHTKPHVHTK); T7: toll-like receptor-7 agonist; Ad-MAGE-1, recombinant adenovirus encoding MAGE-1; MFC Ag, murine forestomach carcinoma antigen; MFC, murine forestomach carcinoma; MG7-Ag, MG7 antigen; NS, not specified; WT-1, Wilms tumor protein-1.

**Table 8:** Results of in vivo vaccinations.

Study	Groups	Vaccination Cytotoxic Effect	Effect on Tumor Size	Effect on Survival
Liu [28]	Group A: pulsed DCs Group B: unpulsed DCs Group C: Control group	Highest in Group A	Tumor size at day 21 in Group A vs. Group B vs. Group C: 0.3688 vs. 0.7536 vs. 2.6323	NS *
Wang [29]	Group A: T7-MG1 Group B: T7-MG3 Group C: PBS control	Group B vs. Group C: 40.92% vs. 16.29% (*p* < 0.01)	37.36% weight reduction in Group B vs. Group C (*p* < 0.01)	NS **
He [30]	Study Group: DC-Ad-MAGE-1 Adenoviral control Group: DC-Ad-LacZ Tumor lysate Group: DC-MFC Ag Control Group: Untreated DCs	Higher in the Study Group vs. Controls	Significantly reduced in the Study Group (*p* < 0.05)	OS > 60 days for 50% of mice
Nagaoka [31]	mCdt1 Group mScarb2 Group mZfp106 Group Control group (untreated)	Highest in the mCdt1 Group	Significantly reduced in the mCdt1 Group vs. Control group (*p* < 0.0001)	NS
Zhu [16]	Study Group: DC-MG-7Ag Negative control group: lentiviral vector without MG-7Ag Control group: untreated DCs	Stronger in the Study Group vs. Control Groups	Significantly reduced in the Study Group	NS
Lu [33]	Study Group: WT1-pulsed DCs Control Group: no DC vaccine	Increased in the Study Group	No difference	Patients with stable disease had longer survival than those with progressive disease (*p* < 0.05)

DCs, dendritic cells; CTLs, cytotoxic T lymphocyte; NS, not specified; MG1: monoclonal gastric cancer 7 antigen mono-epitope (peptide sequence is KPHVHTK); MG3: monoclonal gastric cancer 7 antigen tri-epitope (peptide sequence is KPHVHTKPHVHTKPHVHTK); T7: toll-like receptor-7 agonist; Ad-MAGE-1, recombinant adenovirus encoding MAGE-1; MFC Ag, murine forestomach carcinoma antigen; MFC, murine forestomach carcinoma; MG-7Ag, MG7 antigen; WT-1, Wilms tumor protein-1. * Potential survival benefit of the pulsed DCs group compared to the control. ** Potential survival benefit of the T7-MG3 group compared to the control.

## Data Availability

Data supporting the reported results are available from the corresponding authors upon request.

## References

[1] Bray F., Ferlay J., Soerjomataram I., Siegel R.L., Torre L.A., Jemal A. (2018). Global cancer statistics 2018: GLOBOCAN estimates of incidence and mortality worldwide for 36 cancers in 185 countries. CA A Cancer J. Clin..

[2] Zhao Q., Cao L., Guan L., Bie L., Wang S., Xie B., Chen X., Shen X., Cao F. (2019). Immunotherapy for gastric cancer: Dilemmas and prospect. Brief. Funct. Genom..

[3] Zhao Y., Bai Y., Shen M., Li Y. (2022). Therapeutic strategies for gastric cancer targeting immune cells: Future directions. Front. Immunol..

[4] Bass A.J., Thorsson V., Shmulevich I., Reynolds S.M., Miller M., Bernard B., Hinoue T., Laird P.W., Curtis C., The Cancer Genome Atlas Research Network (2014). Comprehensive molecular characterization of gastric adenocarcinoma. Nature.

[5] Alsina M., Moehler M., Hierro C., Guardeño R., Tabernero J. (2016). Immunotherapy for Gastric Cancer: A Focus on Immune Checkpoints. Target. Oncol..

[6] Hu Z., Ott P.A., Wu C.J. (2018). Towards personalized, tumour-specific, therapeutic vaccines for cancer. Nat. Rev. Immunol..

[7] Morad G., Helmink B.A., Sharma P., Wargo J.A. (2021). Hallmarks of response, resistance, and toxicity to immune checkpoint blockade. Cell.

[8] Smith J.P., Cao H., Chen W., Mahmood K., Phillips T., Sutton L., Cato A. (2021). Gastrin Vaccine Alone and in Combination with an Immune Checkpoint Antibody Inhibits Growth and Metastases of Gastric Cancer. Front. Oncol..

[9] Restifo N.P., Dudley M.E., Rosenberg S.A. (2012). Adoptive immunotherapy for cancer: Harnessing the T cell response. Nat. Rev. Immunol..

[10] Cheever M.A., Higano C.S. (2011). PROVENGE (Sipuleucel-T) in Prostate Cancer: The First FDA-Approved Therapeutic Cancer Vaccine. Clin. Cancer Res..

[11] Kantoff P.W., Higano C.S., Shore N.D., Berger E.R., Small E.J., Penson D.F., Redfern C.H., Ferrari A.C., Dreicer R., Sims R.B. (2010). Sipuleucel-T Immunotherapy for Castration-Resistant Prostate Cancer. N. Engl. J. Med..

[12] Yu J.S., Liu G., Ying H., Yong W.H., Black K.L., Wheeler C.J. (2004). Vaccination with Tumor Lysate-Pulsed Dendritic Cells Elicits Antigen-Specific, Cytotoxic T-Cells in Patients with Malignant Glioma. Cancer Res..

[13] Chan T., Sami A., El-Gayed A., Guo X., Xiang J. (2006). HER-2/neu-gene engineered dendritic cell vaccine stimulates stronger HER-2/neu-specific immune responses compared to DNA vaccination. Gene Ther..

[14] Huarte E., Cubillos-Ruiz J.R., Nesbeth Y.C., Scarlett U.K., Martinez D.G., Buckanovich R.J., Benencia F., Stan R.V., Keler T., Sarobe P. (2008). Depletion of Dendritic Cells Delays Ovarian Cancer Progression by Boosting Antitumor Immunity. Cancer Res..

[15] Banchereau J., Steinman R.M. (1998). Dendritic cells and the control of immunity. Nature.

[16] Zhu B., Sun Y., Wei X., Zhou H., Cao J., Li C., Wu N. (2022). Dendritic Cell Vaccine Loaded with MG-7 Antigen Induces Cytotoxic T Lymphocyte Responses Against Gastric Cancer. J. Healthc. Eng..

[17] Yu Z., Restifo N.P. (2002). Cancer vaccines: Progress reveals new complexities. J. Clin. Investig..

[18] Overwijk W.W., Lee D.S., Surman D.R., Irvine K.R., Touloukian C.E., Chan C.C., Carroll M.W., Moss B., Rosenberg S.A., Restifo N.P. (1999). Vaccination with a recombinant vaccinia virus encoding a “self” antigen induces autoimmune vitiligo and tumor cell destruction in mice: Requirement for CD4^+^ T lymphocytes. Proc. Natl. Acad. Sci. USA.

[19] Van Elsas A., Hurwitz A.A., Allison J.P. (1999). Combination Immunotherapy of B16 Melanoma Using Anti–Cytotoxic T Lymphocyte–Associated Antigen 4 (Ctla-4) and Granulocyte/Macrophage Colony-Stimulating Factor (Gm-Csf)-Producing Vaccines Induces Rejection of Subcutaneous and Metastatic Tumors Accompanied by Autoimmune Depigmentation. J. Exp. Med..

[20] Rosenberg S.A., Yang J.C., Schwartzentruber D.J., Hwu P., Marincola F.M., Topalian S.L., Restifo N.P., Dudley M.E., Schwarz S.L., Spiess P.J. (1998). Immunologic and therapeutic evaluation of a synthetic peptide vaccine for the treatment of patients with metastatic melanoma. Nat. Med..

[21] Page M.J., McKenzie J.E., Bossuyt P.M., Boutron I., Hoffmann T.C., Mulrow C.D., Shamseer L., Tetzlaff J.M., Akl E.A., Brennan S.E. (2021). The PRISMA 2020 statement: An updated guideline for reporting systematic reviews. BMJ.

[22] Hooijmans C.R., Rovers M.M., De Vries R.B., Leenaars M., Ritskes-Hoitinga M., Langendam M.W. (2014). SYRCLE’s risk of bias tool for animal studies. BMC Med. Res. Methodol..

[23] Macleod M.R., O’Collins T., Howells D.W., Donnan G.A. (2004). Pooling of Animal Experimental Data Reveals Influence of Study Design and Publication Bias. Stroke.

[24] Cai Y.G., Fang D.C., Chen L., Tang X.D., Chen T., Yu S.T., Luo Y.H., Xiong Z., Wang D.X., Yang S.M. (2007). Dendritic Cells Reconstituted with a Human Heparanase Gene Induce Potent Cytotoxic T-Cell Responses Against Gastric Tumor Cells In Vitro. Tumor Biol..

[25] Song Z., Guo C., Li Y., Tan B., Fan L., Xiao J. (2012). Enhanced antitumor effects of a dendritic cell vaccine transfected with gastric cancer cell total RNA carrying the 4-1BBL gene in vitro. Exp. Ther. Med..

[26] Iwauchi T., Tanaka H., Yamazoe S., Yashiro M., Yoshii M., Kubo N., Muguruma K., Sawada T., Ohira M., Hirakawa K. (2011). Identification of HLA-A*2402-restricted epitope peptide derived from ERas oncogene expressed in human scirrhous gastric cancer. Cancer Sci..

[27] Kohnepoushi C., Nejati V., Delirezh N., Biparva P. (2019). Poly Lactic-co-Glycolic Acid Nanoparticles Containing Human Gastric Tumor Lysates as Antigen Delivery Vehicles for Dendritic Cell-Based Antitumor Immunotherapy. Immunol. Investig..

[28] Liu B.Y. (2004). Antitumor effects of vaccine consisting of dendritic cells pulsed with tumor RNA from gastric cancer. World J. Gastroenterol..

[29] Wang X.D. (2015). Conjugation of toll-like receptor-7 agonist to gastric cancer antigen MG7-Ag exerts antitumor effects. World J. Gastroenterol..

[30] He S., Wang L., Wu Y., Li D., Zhang Y. (2010). CCL3 and CCL20-recruited dendritic cells modified by melanoma antigen gene-1 induce anti-tumor immunity against gastric cancer ex vivo and in vivo. J. Exp. Clin. Cancer Res..

[31] Nagaoka K., Sun C., Kobayashi Y., Kanaseki T., Tokita S., Komatsu T., Maejima K., Futami J., Nomura S., Udaka K. (2021). Identification of Neoantigens in Two Murine Gastric Cancer Cell Lines Leading to the Neoantigen-Based Immunotherapy. Cancers.

[32] Nabeta Y., Sahara H., Suzuki K., Kondo H., Nagata M., Hirohashi Y., Sato Y., Wada Y., Sato T., Wada T. (2000). Induction of Cytotoxic T Lymphocytes from Peripheral Blood of Human Histocompatibility Antigen (HLA)-A31^+^ Gastric Cancer Patients by In Vitro Stimulation with Antigenic Peptide of Signet Ring Cell Carcinoma. Jpn. J. Cancer Res..

[33] Lu X., Liu J., Cui P., Liu T., Piao C., Xu X., Zhang Q., Xiao M., Liu X., Wang Y. (2018). Co-inhibition of TIGIT, PD1, and Tim3 reverses dysfunction of Wilms tumor protein-1 (WT1)-specific CD8+ T lymphocytes after dendritic cell vaccination in gastric cancer. Am. J. Cancer Res..

[34] Westdorp H., Sköld A.E., Snijer B.A., Franik S., Mulder S.F., Major P.P., Foley R., Gerritsen W.R., De Vries I.J.M. (2014). Immunotherapy for Prostate Cancer: Lessons from Responses to Tumor-Associated Antigens. Front. Immunol..

[35] Butts C., Maksymiuk A., Goss G., Soulières D., Marshall E., Cormier Y., Ellis P.M., Price A., Sawhney R., Beier F. (2011). Updated survival analysis in patients with stage IIIB or IV non-small-cell lung cancer receiving BLP25 liposome vaccine (L-BLP25): Phase IIB randomized, multicenter, open-label trial. J. Cancer Res. Clin. Oncol..

[36] Tobias J., Garner-Spitzer E., Drinić M., Wiedermann U. (2022). Vaccination against Her-2/neu, with focus on peptide-based vaccines. ESMO Open.

[37] Elrifai W., Powell S. (2002). Molecular biology of gastric cancer*. Semin. Radiat. Oncol..

[38] Neek M., Kim T.I., Wang S.W. (2019). Protein-based nanoparticles in cancer vaccine development. Nanomed. Nanotechnol. Biol. Med..

[39] Dobrovolskienė N., Pašukonienė V., Darinskas A., Kraśko J.A., Žilionytė K., Mlynska A., Gudlevičienė Ž., Mišeikytė-Kaubrienė E., Schijns V., Lubitz W. (2018). Tumor lysate-loaded Bacterial Ghosts as a tool for optimized production of therapeutic dendritic cell-based cancer vaccines. Vaccine.

[40] Wei F.Q., Sun W., Wong T.S., Gao W., Wen Y.H., Wei J.W., Wei Y., Wen W.P. (2016). Eliciting cytotoxic T lymphocytes against human laryngeal cancer-derived antigens: Evaluation of dendritic cells pulsed with a heat-treated tumor lysate and other antigen-loading strategies for dendritic-cell-based vaccination. J. Exp. Clin. Cancer Res..

[41] Papież M.A., Krzyściak W. (2021). Biological Therapies in the Treatment of Cancer—Update and New Directions. Int. J. Mol. Sci..

[42] Mao M., Liu S., Zhou Y., Wang G., Deng J., Tian L. (2020). Nanostructured lipid carrier delivering chlorins e6 as in situ dendritic cell vaccine for immunotherapy of gastric cancer. J. Mater. Res..

[43] Draube A., Klein-González N., Mattheus S., Brillant C., Hellmich M., Engert A., Von Bergwelt-Baildon M. (2011). Dendritic Cell Based Tumor Vaccination in Prostate and Renal Cell Cancer: A Systematic Review and Meta-Analysis. PLoS ONE.

[44] Calvo Tardón M., Allard M., Dutoit V., Dietrich P.Y., Walker P.R. (2019). Peptides as cancer vaccines. Curr. Opin. Pharmacol..

[45] Bowen W.S., Svrivastava A.K., Batra L., Barsoumian H., Shirwan H. (2018). Current challenges for cancer vaccine adjuvant development. Expert. Rev. Vaccines.

[46] Giuppi M., La Salvia A., Evangelista J., Ghidini M. (2021). The Role and Expression of Angiogenesis-Related miRNAs in Gastric Cancer. Biology.

[47] Wei Y., Gao L., Yang X., Xiang X., Yi C. (2022). Inflammation-Related Genes Serve as Prognostic Biomarkers and Involve in Immunosuppressive Microenvironment to Promote Gastric Cancer Progression. Front. Med..

[48] Zheng Y., Wu S., Huang X., Luo L. (2023). Ferroptosis-Related lncRNAs Act as Novel Prognostic Biomarkers in the Gastric Adenocarcinoma Microenvironment, Immunotherapy, and Chemotherapy. Oxidative Med. Cell. Longev..

[49] Yuan M., Li X., Song X., Chen X., Wang Y., Han S., Ni Y., Liu D. (2025). Lactate metabolism-related genes serve as potential biomarkers for predicting gastric cancer progression and immunotherapy. Discov. Oncol..

[50] Cheng Z., Lu J., Chen Y., Cao W., Shao Q. (2025). The role of CD101 and Tim3 in the immune microenvironment of gastric cancer and their potential as prognostic biomarkers. Int. Immunopharmacol..

[51] Xu H., Huang K., Lin Y., Gong H., Ma X., Zhang D. (2023). Glycosyltransferase GLT8D1 and GLT8D2 serve as potential prognostic biomarkers correlated with Tumor Immunity in Gastric Cancer. BMC Med. Genom..

